# The CBP-1/p300 Lysine Acetyltransferase Regulates the Heat Shock Response in *C. elegans*

**DOI:** 10.3389/fragi.2022.861761

**Published:** 2022-04-27

**Authors:** Lindsey N. Barrett, Sandy D. Westerheide

**Affiliations:** University of South Florida, Department of Cell Biology, Microbiology, and Molecular Biology, Tampa, FL, United States

**Keywords:** HSF-1, CBP-1, heat shock response, aging, *C. elegans*

## Abstract

The decline of proteostasis is a hallmark of aging that is, in part, affected by the dysregulation of the heat shock response (HSR), a highly conserved cellular response to proteotoxic stress in the cell. The heat shock transcription factor HSF-1 is well-studied as a key regulator of proteostasis, but mechanisms that could be used to modulate HSF-1 function to enhance proteostasis during aging are largely unknown. In this study, we examined lysine acetyltransferase regulation of the HSR and HSF-1 in *C. elegans*. We performed an RNA interference screen of lysine acetyltransferases and examined mRNA expression of the heat-shock inducible gene *hsp-16.2*, a widely used marker for HSR activation. From this screen, we identified one acetyltransferase, CBP-1, the *C. elegans* homolog of mammalian CREB-binding protein CBP/p300, as a negative regulator of the HSR. We found that while knockdown of CBP-1 decreases the overall lifespan of the worm, it also enhances heat shock protein production upon heat shock and increases thermotolerance of the worm in an HSF-1 dependent manner. Similarly, we examined a hallmark of HSF-1 activation, the formation of nuclear stress bodies (nSBs). In analyzing the recovery rate of nSBs, we found that knockdown of CBP-1 enhanced the recovery and resolution of nSBs after stress. Collectively, our studies demonstrate a role of CBP-1 as a negative regulator of HSF-1 activity and its physiological effects at the organismal level upon stress.

## Introduction

One of the hallmarks of aging is the dysregulation of protein homeostasis, or proteostasis, in the cell which is critical for cell survival and function (López-Otín et al., 2013). The heat shock response (HSR) is a key player in the proteostasis network, known to play a role in a variety of physiological processes, including development, reproduction, aging, and age-related diseases (Labbadia and Morimoto, 2015; Joutsen and Sistonen, 2019). The HSR is a highly conserved cellular response that maintains proteostasis in the cell through the activation of heat shock transcription factor 1 (HSF1). HSF1 is the master regulator of the HSR and when activated, drives the expression of heat shock proteins (HSPs) that act as molecular chaperones to restore proteostasis after stress (Hartl et al., 2011). HSF1 has been implicated in a wide array of age-related diseases, including neurodegenerative diseases, metabolic diseases, and cancer (Powers et al., 2009; Gomez-Pastor et al., 2017). Understanding HSF1 regulation with aging may be useful when developing therapeutic strategies for such diseases.

The transcriptional activity of HSF1 is stress-inducible and highly regulated (Westerheide et al., 2012). Human HSF1 is regulated in part through post-translational modifications. These modifications include acetylation, phosphorylation, and sumoylation, and they function in both the activation and attenuation of HSF1 (Gomez-Pastor et al., 2017). Studies have shown multiple site-specific, reversible acetylation modifications on HSF1 that affect its overall protein stability and attenuation (Westerheide et al., 2009; Raychaudhuri et al., 2014; Zelin and Freeman, 2015). Under non-stress conditions, HSF1 levels are stabilized by the acetylation of Lys208 and Lys298 by the lysine acetyltransferase (KAT) CBP/p300 (Raychaudhuri et al., 2014). During stress, CBP/p300 also mediates the acetylation of Lys80 of HSF1, which aids in inhibiting HSF1 binding to DNA to attenuate the HSR (Westerheide et al., 2009; Raychaudhuri et al., 2014). There are also multiple other known sites of HSF1 acetylation where the effect of acetylation has yet to be elucidated.

The activity of HSF-1, the *C. elegans* homolog of HSF1, is highly conserved and makes *C. elegans* a useful model organism to study the regulation of HSF-1 during stress and aging. HSF-1 has been shown to interact with CREB binding protein CBP-1, the *C. elegans* homolog of CBP/p300, in lifespan regulation (Zhang et al., 2009); however, the mechanism by which CBP-1 regulates HSF-1 has not been examined.

CBP-1 in *C. elegans* functions as a chromatin remodeler and lysine acetyltransferase that can regulate transcription (Victor et al., 2002). CBP-1 is an essential protein for embryonic development, cell differentiation, and aging, and knockdown of *cbp-1* significantly reduces lifespan (Shi and Mello 1998; Victor et al., 2002; Zhang et al., 2009; Cai et al., 2019). CBP-1 also acts in multiple stress response pathways, including the mitochondrial unfolded protein response and the oxidative stress response (Ganner et al., 2019; Li et al., 2021). Although CBP-1 interactions with HSF-1 have been described, its role, as well as the potential role of other lysine acetyltransferases, in regulating HSF-1 and the HSR are still unknown.

In this study, we utilized *C. elegans* to screen for lysine acetyltransferase modulators of the HSR. We identified a role of CBP-1 in negatively regulating the HSR in *C. elegans*, confirming the importance of this regulator in this stress response pathway. Collectively, our studies demonstrate the ability of CBP-1 to regulate the HSR in an HSF-1 dependent-manner and highlight its effects on stress resistance and longevity.

## Methods

### *C. elegans* Strains and Maintenance

The following strains were used in this study: N2 (Bristol), PS3551–*hsf-1* (sy441), MH2430—*cbp-1* (ku258), SDW015–*hsf-1* (asd002 (*hsf-1:GFP* + *unc-119* (+)), and SDW173. The MH2430 strain was outcrossed three times to the N2 wildtype strain to generate the SDW173 strain. Strains were maintained at 20°C on standard NGM plates seeded with *Escherichia coli* OP50-1. The synchronous population of nematodes was obtained by bleach synchronization and plated for 19 h on NGM plates without food.

### RNA Interference

Synchronous L1 nematodes were grown on standard NGM plates seeded with OP50-1 bacteria for 19 h to prevent potential RNAi-mediated effects on early development. Worms were transferred onto standard NGM plates supplemented with 25 μg/ml carbenicillin and 1 mM isopropyl-beta-D-thiogalactopyranoside (IPTG) and seeded with either HT115 bacteria containing an empty vector (EV/L4440 control) or with sequence-verified gene-specific RNAi strains isolated from the Ahringer RNAi library (J. Ahringer, University of Cambridge, Cambridge, U.K.), as previously described (Kamath et al., 2003). To induce dsRNA production, HT115 bacteria were supplemented with 1 mM IPTG shaking at 37°C for 1 h before seeding.

### RNA Isolation and Quantitative PCR

RNA was extracted with TRIzol^®^ reagent (Ambion^®^, cat# 15,596–026) by standard protocol. RNA was reverse-transcribed using a High Capacity cDNA Reverse Transcription Kit (Applied Biosystems, cat# 4,368,814). cDNA was diluted to 100 ng/μl to be used as a template for qRT–PCR performed with the StepOne Plus Real-time PCR system (Applied Biosystems, cat # 4,376,600) using iTaq™ Universal SYBR^®^ Green Supermix (Bio-Rad, cat# 1,725,121) according to the manufacturer’s instructions. Expression levels were analyzed *via* qPCR using the ΔΔCt method. The housekeeping gene *cdc-42* (R07G3.1) was used for normalization. Results show averages of independent biological triplicates performed in technical triplicates. Statistical analysis was performed with GraphPad (GraphPad Software, https://www.graphpad.com) using an unpaired Student’s t-test.

Primers used: *hsp16.2* (Y46H3A.3) Fwd: ACG​CCA​ATT​TGC​TCC​AGT​CT, Rvs: TGA​TGG​CAA​ACT​TTT​GAT​CAT​TGT; *hsp-70* (C12C8.1) Fwd: TTC​AAT​GGG​AAG​GAC​CTC​AAC​T, Rvs: GGC​TGC​ACC​AAA​GGC​TAC​TG; *cdc-42* (R07G3.1) Fwd: CTT​CTG​AGT​ATG​TGC​CGA​CAG​TCT, Rvs: GGCTCGCCACCGATCAT; *hsp16.48* (T27E4.3) Fwd: TTG​GAG​AAA​TGC​TGA​TCA​CAA​CTC, Rvs: TTT​TTA​GTT​CTC​TTC​CAT​CCA​ATT​CA; F44E5.5 Fwd: CTT​CAT​GCA​AAG​CTA​TTG​GTA​TCG, Rvs: CTT​CCG​AGT​TGG​CGA​GGA​T; *cbp-1* (R10E11.1) Fwd: GCA​GCG​AAA​ACG​GAG​GAA, Rvs: GCA​TGG​AAC​AAA​TGT​GGA​GTC​TT; *hsf-1* (Y53C10A.12) Fwd: TGC​AGC​CAG​GAT​TGT​CGA, Rvs: GGC​GGC​GCA​AAA​GTC​TAT​T; *hat-1* (M03C11.4) Fwd: ACG​GAC​TTG​CTG​TCG​TTA​AA, Rvs: CCG​AAG​ATT​GTC​TCC​TCA​TCT​C; *kat-1* (T02G5.8) Fwd: CCA​CAT​CTG​CTG​CAC​TAT​CA, Rvs: GCA​GTT​ACC​GAA​GAG​AGA​GAA​G; *taf-1* (W04A8.7) Fwd: TAC​GAG​GCC​ACA​GCT​TAT​TG, Rvs: CGC​TTC​TCC​TCC​TTA​TAC​TGT​TC; *mys-1* (VC5.4) Fwd: CGA​GCT​GCA​AAT​GGT​TCA​ATT​A, Rvs: GTA​GCT​CAC​ACG​ACG​CTA​AA; *mys-2* (K03D10.3) Fwd: GGA​GCG​AAA​GAG​CTC​ATG​TAT​T; Rvs: GCT​CGA​CTA​CCA​CTT​CGT​TTA​C; *mec-17* (F57H12.7) Fwd: GGT​CAT​CAG​AGC​AAG​GGA​AA, Rvs: TTG​GCC​TGA​TGA​GCT​CTA​TTG; *atat-2* (W06B11.1) Fwd: GTT​CAG​CTG​TGT​CCA​GTC​AT, Rvs: TAA​CAT​ATC​CTG​GCG​CAT​CAA; T02G5.4 Fwd: GCA​GTT​ACC​GAA​GAG​AGA​GAA​G, Rvs: CCA​CAT​CTG​CTG​CAC​TAT​CA; *hsp-1* (F26D10.3) Fwd: TCA​AGA​GAA​ACA​CCA​CCA​TCC, Rvs: GGC​ACG​TTC​TCC​TTC​GTA​AA; *hsp-90* (C47E8.5) Fwd: AGT​ACT​GCG​TCC​AAC​AAC​TC, Rvs: TCT​TCT​CCT​CCT​CGG​TTT​CT.

### Thermotolerance Analysis

Thermotolerance was tested by exposing Day 1 animals to 37°C heat shock for 3.5 h and then determining survival 48 h later by assessing response to a gentle touch. For each trial, ∼ 100 randomly selected individual animals were assessed for the fraction alive (live/total). Thermotolerance assay data reflects three biologically independent trials. Data was plotted as the % survival using GraphPad Prism (GraphPad Software, https://www.graphpad.com) and was analyzed with a two-tailed *t* test.

### Lifespan Analysis

All lifespan assays were performed at 20°C with approximately 100 worms per condition in biological triplicate. Animals were transferred to fresh plates daily for 5 days to avoid progeny contamination. Adult worms were scored approximately every day and counted as dead when no response was observed by poking with a platinum wire. The average survivability of three replicates was plotted using OASIS 2 (https://sbi.postech.ac.kr/oasis2/) (Han et al., 2016), and statistical analysis was done by Log-rank (Mantel-Cox) Test.

### Fluorescent Microscopy and Nuclear Stress Body Assessment

Fluorescent images were obtained using a Keyence BZ-X fluorescent microscope. Animals were picked free of bacteria and anesthetized with 10 mM levamisole. Nuclear stress body formation was quantified by assessing for the presence of nuclear foci containing HSF-1:GFP in hypodermal cells. The heat shock conditions for nuclear stress body assessment were 15 min in a 33°C water bath on plates wrapped in parafilm. After anesthetizing and placing the cover slip on top of the worms, they were imaged within 10–15 min to avoid the formation of nuclear stress bodies which may be due to hypoxia or other cytotoxic stress (*n* = ∼ 10 worms per condition). Quantification was performed in GraphPad Prism (GraphPad Software, www.graphpad.com) and **s**ignificance was determined using 2-way ANOVA.

### Statistical Analyses

Statistical analyses were carried out with GraphPad Software (GraphPad Software, La Jolla, CA, United States, https://www.graphpad.com) unless otherwise stated. All error bars are representative of standard deviation between independent biological replicates, as indicated.

## Results

### Targeted-RNAi Screen for Lysine Acetyltransferase Modulators of the HSR Identifies CBP-1

**FIGURE 1:**
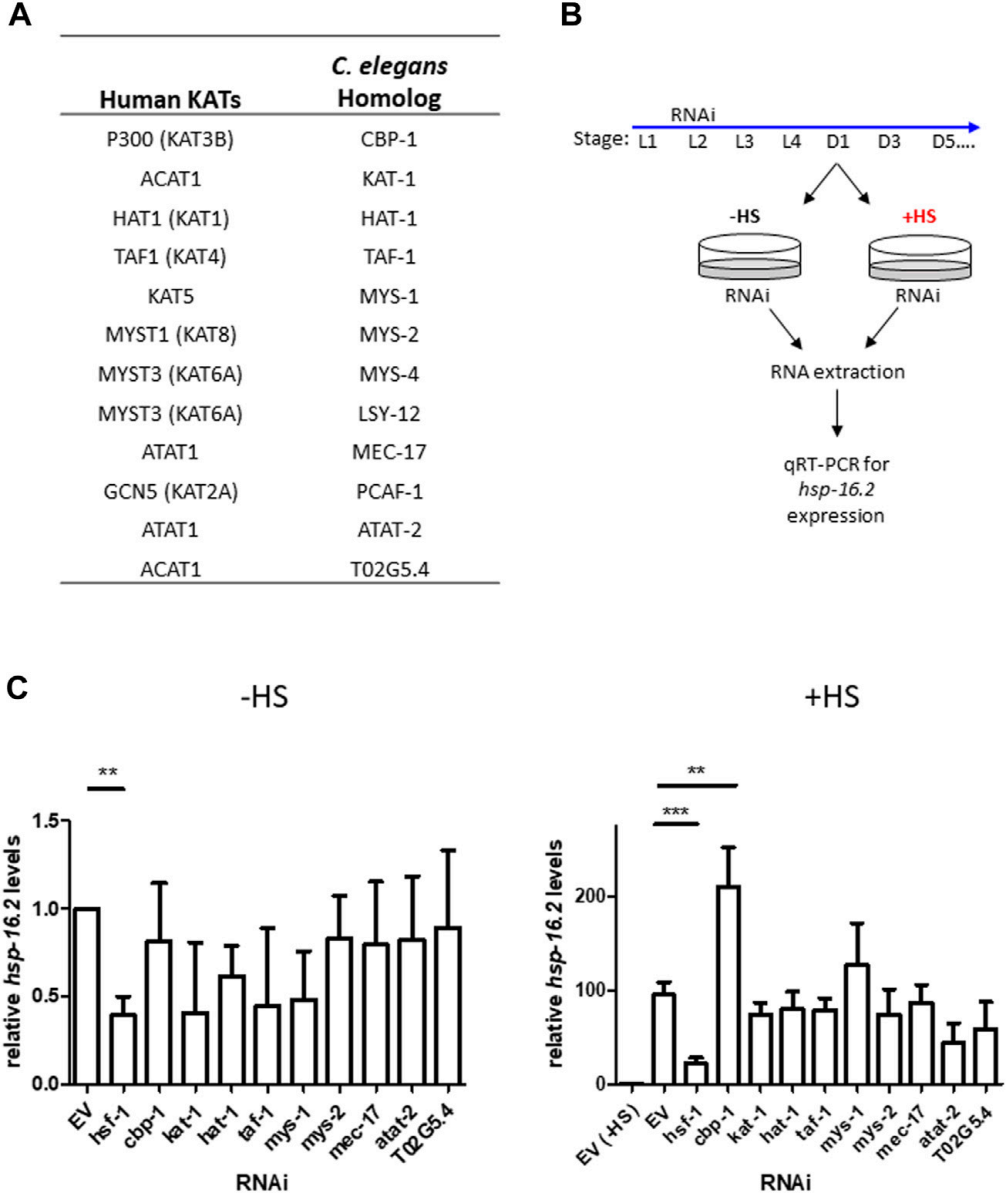
Targeted-RNAi screen for lysine acetyltransferase modulators of the HSR identifies CBP-1. **(A)** Table of all putative lysine acetyltransferases (KATs) and their human homologs. **(B)** Schematic of the targeted RNAi screen for lysine acetyltransferase modulators of the HSR. ∼ 75% of worm KATs were screened by examining endogenous *hsp-16.2 gene expression*, a heat-inducible heat shock protein gene, before and after heat shock using qRT-PCR. **(C)** N2 (wildtype) animals were fed empty vector (EV) control or KAT RNAi from 19 h after L1 larval stage to Day 1 of adulthood. RNA was extracted from Day 1 animals before and immediately after a 1-h heat shock at 33°C and *hsp-16.2* levels were quantified by qRT-PCR using the ΔΔCt method (*n* = 3 biologically independent samples). The housekeeping gene cdc-42 (R07G3.1) was used for normalization. Statistical analysis was determined by conducting a One-Way ANOVA using GraphPad Prism (GraphPad Software, www.graphpad.com) followed by a Tukey post hoc test comparison of all columns. *p-value < 0.05, **p-value < 0.01, ***p-value < 0.001.

To identify lysine acetyltransferases that may regulate the HSR, we performed an RNA interference (RNAi) screen by targeting ∼ 75% of all putative lysine acetyltransferases (KATs) in *C. elegans* using RNAi knockdown (Figure 1A) *C. elegans* KATs were identified by searching the *C. elegans* protein database for proteins containing conserved acetyltransferase domains to those of known human KATs (Sheikh and Akhtar, 2019). We utilized quantitative RT-PCR to assess the heat shock inducibility of *hsp-16.2* mRNA expression, a widely used marker of HSR activity, after KAT RNAi knockdown (Golden et al., 2020). We induced RNAi 19 h after the L1 stage until Day 1 of adulthood, when we extracted RNA before and immediately after a 1-h heat shock at 33°C to assess *hsp-16.2* expression (Figure 1B). Our KAT and *hsf-1* RNAi conditions suppress expression of their corresponding genes by about 50% (Supplementary Figure S1). From this screen, we found no significant changes in *hsp-16.2* expression in non-heat shock conditions after KAT RNAi knockdown (Figure 1C). After heat shock, only RNAi for *cbp-1* (R10E11.1), the homolog of human CBP/p300 CREB binding protein, significantly altered the mRNA expression of *hsp16.2* expression, resulting in an increase in expression of approximately 2-fold (Figure 1C).

### CBP-1 Regulates the Expression of Multiple HSF-1 Target Genes in an HSF-1-Dependent Manner

**FIGURE 2:**
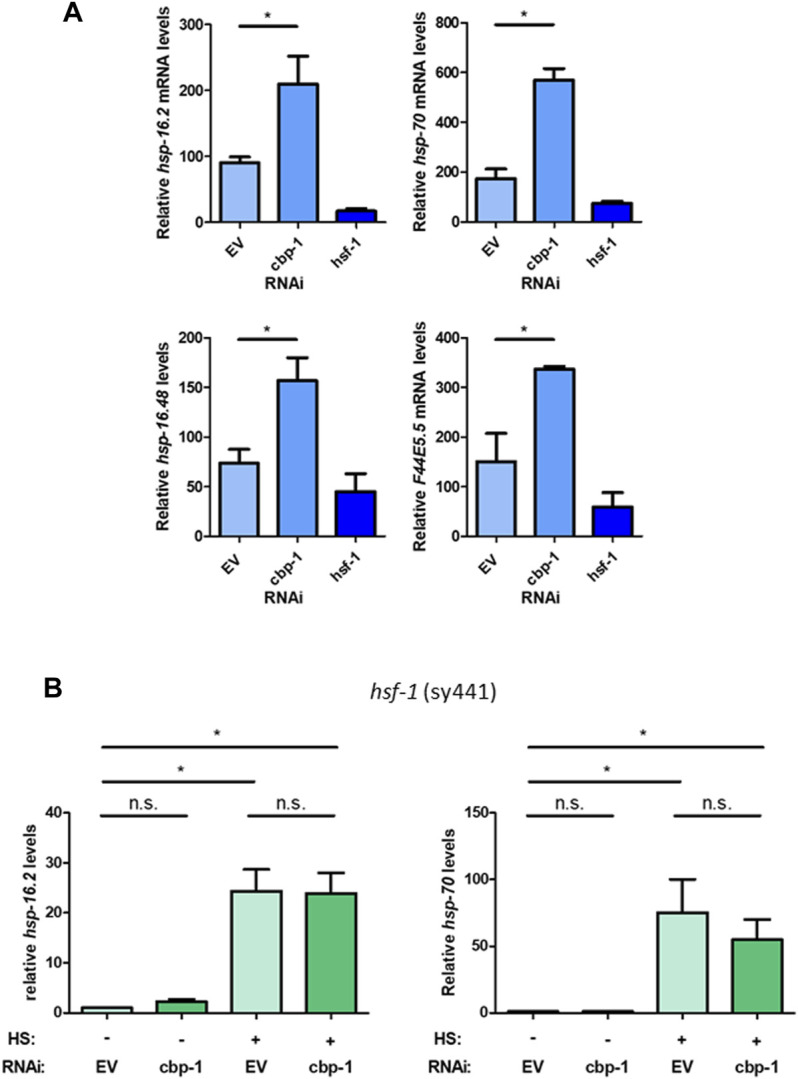
CBP-1 regulates the expression of multiple HSF-1 target genes in an HSF-1-dependent manner. **(A)** RNA was extracted from N2 (wildtype) worms on Day 1 of adulthood with a 1-h heat shock at 33°C that were fed empty vector (EV), *cbp-1,* or *hsf-1* RNAi beginning 19 h after L1. Heat shock inducible heat shock protein gene expression levels for *hsp-16.2, hsp-16.48, F44E5.5,* and *hsp-70* were quantified by qRT-PCR. **(B)** RNA was extracted from an HSF-1 deletion worm strain (PS3551) on Day 1 of adulthood with and without a 1-h heat shock at 33°C with animals fed EV or *cbp-1* RNAi. PS3551 animals were fed empty vector (EV) control or *cbp-1* RNAi from 19 h after L1 larval stage to Day 1 of adulthood. Heat shock inducible *hsp-16.2* and *hsp-70* levels were quantified by qRT-PCR. Statistical analysis was determined by determined by conducting a One-Way ANOVA using GraphPad Prism (GraphPad Software, www.graphpad.com) followed by a Tukey post hoc test comparison of all columns. *p-value < 0.05.

To further examine the role of CBP-1 on the HSR, we examined how knockdown of *cbp-*1 affects various heat shock inducible HSF-1 target genes using qRT-PCR. The target genes tested, including *hsp-16.2*, *hsp.16.48, hsp-70,* and F44E5.5 (an inducible *hsp-70* family gene), are highly inducible upon heat shock. We found that while *cbp-*1 RNAi didn’t significantly change the expression of the *hsp* target genes tested under basal conditions (Supplementary Figure S2A), *cbp-1* RNAi did significantly increase the expression of these genes after heat shock as compared to EV control (Figure 2A). We also found that *cbp-1* knockdown does not affect *hsf-1* expression, or vice versa (Supplementary Figure S1A). HSF-1 also regulates the expression of a distinct subset of developmental genes independent of the HSR, including *hsp-1* and *hsp-90* (Li et al., 2016). We found that *cbp-1* knockdown does not affect expression of these HSF-1 target genes (Supplementary Figure S2B). Thus, CBP-1 is negatively regulating HSP but not HSF-1 expression after heat stress.

To determine whether this negative regulation of CBP-1 is mediated directly through HSF-1, we used the PS3551 worm strain containing a non-functional HSF-1 (Zhou et al., 2018). This mutant contains a point mutation in the HR-C domain of HSF-1 that causes the expression of a truncated non-functional HSF-1 mutant. We then measured the expression of *hsp-16.2* and *hsp-70* (C12C8.1) in response to *cbp-1* RNAi (beginning 19 h after L1) before and after heat shock in this mutant to determine dependence on HSF-1. While we found that knockdown of *cbp-1* increased *hsp-16.2* expression after heat shock with wildtype HSF-1 (Figure 1A), that increase is abolished with a nonfunctional HSF-1. Knockdown of *cbp-1* does not significantly increase *hsp-16.2* or *hsp-*70 (*C12C8.*1) expression after heat shock compared to control (Figure 2B). We conclude that CBP-1 depends on HSF-1 in order to regulate *hsp* gene expression.

### Effect of *cbp-1* and *hsf-1* RNAi on Thermotolerance and Lifespan

**FIGURE 3:**
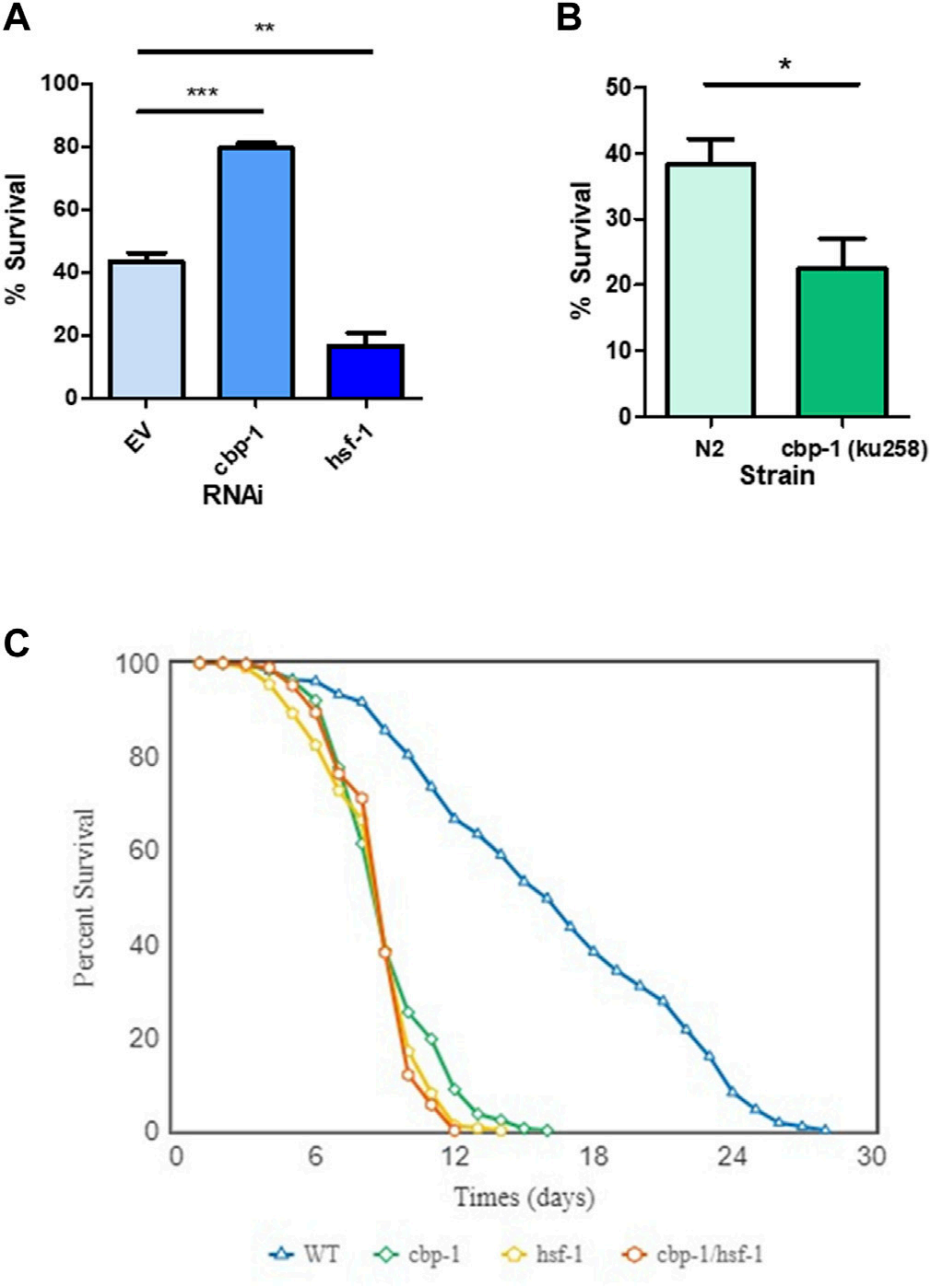
Effect of CBP-1 on thermotolerance and lifespan. **(A)** In order to assess CBP-1 activity on resistance to heat stress, N2 worms were given a lethal heat shock of 3.5 h at 37°C on Day 1 of adulthood and then ∼ 100 animals were randomly scored for survival after a 48-h recovery. Percent survival was determined for worms fed with empty vector (EV), *cbp-1*, and *hsf-1* RNAi. Data was plotted as % survival using GraphPad Prism (GraphPad Software, www.graphpad.com) and was analyzed with a two-tailed *t* test. *p-value < 0.05, **p-value < 0.01, ***p-value < 0.001. **(B)** Thermotolerance was assessed in N2 and SDW173, a strain containing a gain-of-function allele of *cbp-1* that increases acetyltransferase activity (MH2430 strain outcrossed 3x to N2). Data was plotted as % survival using GraphPad Prism (GraphPad Software, www.graphpad.com) and was analyzed with a two-tailed *t* test. *p-value < 0.05. **(C)** Lifespan analysis was performed in wild type N2 animals fed with EV, *cbp-1* RNAi, *hsf-1* RNAi, or *cbp-1/hsf-1* double RNAi throughout the lifespan. Worms were scored approximately every day for survival. The average survivability of three replicates was plotted using OASIS 2 (https://sbi.postech.ac.kr/oasis2/) (Han et al., 2016), and statistical analysis was done by Log-rank (Mantel-Cox) Test (Shown in Supplementary Table S1).

We next wanted to test whether CBP-1 also regulates other physiological readouts of the HSR. To test for resistance to heat stress, we examined thermotolerance, a physiological effect known to be modulated by HSF-1. To test this, we fed the worms *cbp-1*, *hsf-1*, or control RNAi from 19 h after L1 until Day 1 of adulthood prior to subjecting the worms to a lethal heat shock of 37°C for 3.5 h. After a 48-h recovery, the worms were scored alive/dead after a gentle touch. Compared to empty vector control RNAi-treated worms, *hsf-1* RNAi-treated worms had significantly reduced survival after heat shock ( ∼ 20% decrease), as expected (Figure 3A). However, knockdown of *cbp-1* increased the % survival of the worms by ∼ 40% (Figure 3A). Thus, knockdown of *cbp-1* increases thermotolerance.

We then performed a complimentary experiment, assessing how an increase in CBP-1 activity would affect thermotolerance. We utilized the MH3420 strain that contains two point mutations in *cbp-1,* resulting in a gain-of-function allele with increased KAT activity (Eastburn and Han, 2005). This strain was outcrossed three times with our laboratory N2 strain to create the SDW173 strain. N2 and SDW173 worms were synchronized and grown to Day 1 of adulthood then subjected to a lethal heat shock of 37°C for 3.5 h. We found that SDW173 worms had an ∼20% decrease in percent survival as compared to N2 wildtype worms (Figure 3B). This suggests that an increase in CBP-1 acetyltransferase activity decreases thermotolerance. In summary, *cbp-1* RNAi activates thermotolerance, while a *cbp-1* gain-of-function mutant inhibits thermotolerance.

Previous studies have found that *cbp-1* RNAi reduces the lifespan of the worm (Zhang et al., 2009; Cai et al., 2019). We wanted to test whether this effect depends on HSF-1. Worms were fed with EV control, *cbp-1*, *hsf-1*, or *cbp-1/hsf-1* double RNAi from 19 h after L1 throughout their lifespans. The worms were scored approximately every day starting at day 1 of adulthood for survival, and dead worms were scored when non-responsive to poking with a platinum wire. We found that *cbp-1* RNAi decreased lifespan to a significantly lesser extent than *hsf-1* RNAi (Figure 3C). However, the *cbp-1/hsf-1* double RNAi did not further reduce lifespan (Figure 3C).

### *cbp-1* RNAi Increases Recovery Rate of Nuclear Stress Bodies After Heat Shock

**FIGURE 4:**
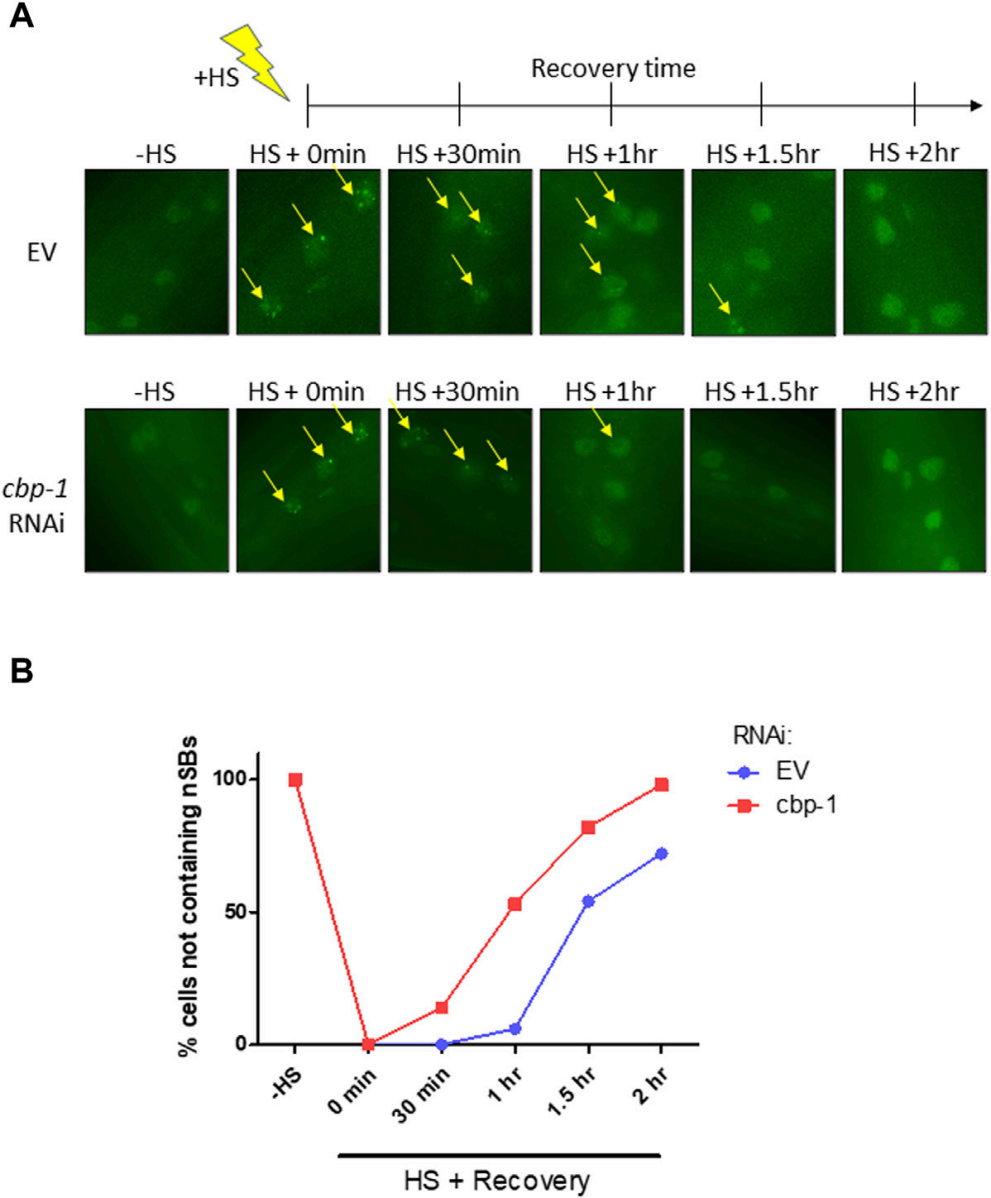
***c*** *bp-1* RNAi increases recovery rate of nuclear stress bodies (nSBs) after heat shock. **(A)** Fluorescent images of HSF-1:GFP in the SDW015 strain were examined before heat shock and in 30-min increments after a 15-min heat shock at 33°C for 2 h at 20°C until the majority of hypodermal cells containing nuclear stress bodies (nSBs) had been resolved. Images represent fluorescent nSB formation in hypodermal cells under control and *cbp-1* RNAi conditions at each time point in Day 2 adult worms. Yellow arrows indicate hypodermal cell nuclei containing nSB foci. **(B)** The percentage of hypodermal cells not containing nSBs were quantitated following fluorescent imaging of worms fed empty vector (EV) control and *cbp-1* RNAi (*n* = 10 worms). Significance was determined using 2-way ANOVA. Column *p-value = 0.0497; row **p-value = 0.0022.

Upon activation of the HSR, HSF-1 undergoes localization changes and forms nuclear stress bodies (nSBs), a hallmark of HSF-1 activation (Deonarine et al., 2021). nSBs require HSF-1 binding and are a sign of activation of the HSR (Morton and Lamitina, 2013). After HSR activation, nSBs form within 5 min of stress detection and gradually dissolve until HSF-1 is diffuse in the nucleus (Figure 4A) (Deonarine et al., 2021). We examined the effect of *cbp-1* RNAi on the rate of nSB recovery after stress. We used the SDW015 (HSF-1:GFP) strain to visualize nSB formation before and after a 15-min heat shock at 33°C on Day 2 of adulthood. We utilized fluorescence microscopy to image hypodermal cells of the worm in 30-min increments until the majority of cells containing nSBs had diffuse HSF-1:GFP expression in the nuclei (Figure 4A). After heat shock, control worms took an average of 1.7 h to recover and resolve their nSBs, whereas worms fed *cbp-1* RNAi took an average of 1.1 h to resolve nSBs (Figure 4B). Thus, knockdown of *cbp-1* significantly increased the rate of recovery of nSBs after heat shock.

## Discussion

Given the importance of post-translational modifications on HSF-1 regulation and function, we sought to elucidate the effect of lysine acetyltransferase regulation on the HSR. Here, we conducted a targeted RNAi screen for lysine acetyltransferase regulation of *hsp-16.2* expression levels and identified CBP-1 as a regulator of *hsp* expression. In addition, *cbp-1* knockdown also increased the expression of multiple other heat shock genes, increased thermotolerance, and improved the recovery rate of nuclear stress bodies after heat stress. Overall, these studies suggest a role for CBP-1 in negatively regulating HSF-1 activity during stress.

CBP-1 is widely known to be a transcriptional coactivator that aids in transcription (Victor et al., 2002). We further examined various other heat-shock inducible *hsp* expression and found that *cbp-1* knockdown increases the expression of multiple *hsp*s (*hsp-16.2, hsp.16.48, hsp-70,* and *F44E5.5*) after heat shock. This type of negative transcriptional regulation is a novel role for CBP-1 in *C. elegans*. However, human CBP/p300 has some characterized instances in which it negatively regulates transcription through effects on transcription factors. For example, acetylation of the elongation factor AFF1 by CBP/p300 inhibits transcription during genotoxic stress (Kumari et al., 2019).

CBP-1 is an essential protein that regulates multiple physiological processes including cell differentiation, embryonic development, metabolism, lifespan, and aging (Shi and Mello, 1998; Zhang et al., 2009; Vora et al., 2013; Cai et al., 2019). Specifically, CBP-1 interacts with DAF-16 and HSF-1 to regulate lifespan during caloric restriction (Zhang et al., 2009). Similarly, our results suggest that CBP-1 and HSF-1 work in the same pathway to regulate lifespan. While our work suggests that CBP-1 negatively regulates the HSR, our lifespan results suggest that it positively affects lifespan and aging. This was unexpected, as activating the HSR generally protects from aging (Hsu et al., 2003; Morley and Morimoto, 2004). Since CBP-1 plays a positive role in a variety of physiological pathways that control aging (Zhang et al., 2009; Cai et al., 2019; Ganner et al., 2019; Zhou et al., 2019; Li et al., 2021) it may be that the effects on these pathways are overriding the effects of CBP on HSF-1 and HSP expression in terms of life span regulation. It will be informative in future work to determine how the regulation of HSF-1 activity by CBP-1 can benefit aging and potentially lifespan in the worm.

While our data indicates that CBP-1 regulates HSF-1 activity, the mechanism of this regulation is still unknown. CBP-1 could directly acetylate HSF-1 as one potential mechanism. Mammalian CBP/p300 acetylates HSF-1 under both stress and non-stress conditions to both stabilize HSF-1 and attenuate HSF-1 from the DNA (Westerheide et al., 2009; Raychaudhuri et al., 2014). CBP/p300 acetylation of HSF1 could be conserved in *C. elegans* and function to attenuate HSF-1 activity. While human CBP/p300 also acts as a scaffolding protein to recruit the transcriptional machinery (Holmqvist and Mannervik, 2013), it is likely that worm CBP-1 regulation of the HSR requires CBP-1 acetyltransferase activity since we found that the SDW173 mutant containing CBP-1 with hyperactive KAT activity decreased thermotolerance. There is also the possibility of CBP-1 regulating HSF-1 through an indirect regulator, as CBP-1 also binds to a multitude of other factors including the stress-responsive factors SKN-1 and DAF-16 (Nasrin et al., 2000; Ganner et al., 2019). Future work to uncover the mechanism behind HSF-1 regulation will allow for a more detailed analysis of the role of CBP-1 during stress and aging.

A hallmark of HSF1 activation in both mammalian cells and *C. elegans* is the formation and resolution of nuclear stress bodies (nSBs) containing HSF1 (Jolly et al., 1999; Jolly et al., 2004; Biamonti and Vourc’h, 2010; Morton and Lamitina, 2013; Deonarine et al., 2021). In mammalian cells, the resolution of nSBs correlates with HSF1 activity, transcription of *hsp* target genes, and cell survival at the single cell level (Gaglia et al., 2020). A recent mammalian study discovered the existence of both HSF1 nSBs at non-*hsp* gene loci and smaller HSF1 condensates at transcriptionally active *hsp* gene loci upon heat shock (Zhang et al., 2022). In this study, HSF1 in the condensates was found to colocalize with transcription apparatus factors, including RNA Pol II, BRD4, MED1, and CYCT1 (Zhang et al., 2022). It is thus possible that upon heat shock, HSF1 in nSBs sequesters transcription factors to halt general transcription, while HSF1 in the smaller condensates functions to induce the transcription of *hsp* target genes. HSF1 nSBs that are not able to resolve may go on to transition from liquid to gel phase condensates, which could then permanently hinder HSF1 activity and the HSR. Our study did not analyze small HSF-1 condensates, and it is not yet known if these are present in the worm. However, we found that *cbp-1* knockdown promotes the recovery of large HSF-1 nSBs after heat shock. Thus, CBP-1 may facilitate the stabilization of HSF-1 nSBs, which could promote the gel phase state upon prolonged stress, leading to ultimate HSF-1 inactivation. We hypothesize that decreasing CBP-1 levels through RNAi could thus enhance the HSR. We do not know how CBP-1 may stabilize HSF-1 nSBs. It could be through the acetylation of HSF-1 or another protein, through providing a scaffolding, or through an alternative mechanism. It will be interesting to investigate these possibilities in future work.

CBP-1 also functions as a regulator of multiple other stress response pathways. Interestingly, CBP-1 positively regulates SKN-1/Nrf activation and the oxidative stress response, where it regulates SKN-1 activity and protein abundance (Ganner et al., 2019). CBP-1 is also an essential regulator of the mitochondrial unfolded protein response (Li et al., 2021). Collectively, our studies identify a new role of CBP-1 in HSR regulation in *C. elegans* and its physiological effects at the organismal level*.* CBP-1 negatively regulates HSP expression, nuclear stress body recovery, and thermotolerance ability of the worm. Future work is needed to determine the precise mechanism of action of CBP-1 in the HSR pathway and any potential acetylation changes of HSF-1 that are induced by CBP-1.

## Data Availability

The original contributions presented in the study are included in the article/Supplementary Material. Further inquiries can be directed to the corresponding author.

## Supplementary Material

Supplementary Figure S1 Validation of KAT and *hsf-1* RNAi. **(A)** RNA was extracted from wildtype (N2) worms on Day 1 of adulthood that were fed empty vector (EV), *cbp-1,* or *hsf-1* RNAi beginning 19 hrs after L1. *cbp-1* and *hsf-1* levels were quantified by qRT-PCR (*n* = 3 biologically independent samples). **(B)** RNA was extracted from N2 worms on Day 1 of adulthood that were fed EV or KAT RNAi beginning 19 hrs after L1. Validation of KAT knockdown was examined by quantifying KAT levels by qRT-PCR (*n* = 3 biologically independent samples). Statistical analysis determined by determined by conducting a One-Way ANOVA using GraphPad Prism (GraphPad Software, www.graphpad.com) followed by a Tukey post hoc test comparison of all columns. *p-value < 0.05, **p-value < 0.01, ***p-value < 0.001.

Supplementary Figure S2
*cbp-1* RNAi does not change the expression of HSF-1 target genes under basal conditions. **(A)** RNA was extracted from N2 (wildtype) worms on Day 1 of adulthood under basal conditions at 20°C that were fed empty vector (EV), *cbp-1,* or *hsf-1* RNAi beginning 19 hrs after L1. Heat shock inducible heat shock protein gene expression levels for *hsp-16.2, hsp-16.48, F44E5.5,* and *hsp-70* were quantified by qRT-PCR. **(B)** RNA was extracted from N2 worms on Day 1 of adulthood under basal conditions that were fed EV or *cbp-1* RNAi beginning 19 hrs after L1. Developmental HSF-1 target genes independent of the heat shock response, *hsp-1* and *hsp-90,* were quantified by qRT-PCR. Statistical analysis was determined by determined by conducting a One-Way ANOVA using GraphPad Prism (GraphPad Software, www.graphpad.com) followed by a Tukey post hoc test comparison of all columns. *p-value < 0.05.

Supplementary Table S1 Lifespan analysis. Analysis of lifespan of EV, *cbp-1, hsf-1,* and *cbp-1/hsf-1* RNAi including mean lifespan and total/censored/scored number of worms per trial (done in biological triplicate). Survivability was plotted using OASIS 2 (https://sbi.postech.ac.kr/oasis2/) (Han *et al.* 2016), and statistical analysis was done by Log-rank (Mantel-Cox) Test.
