# Supplementary figures and images for: The CBP-1/p300 Lysine Acetyltransferase Regulates the Heat Shock Response in C. elegans
Source: Front Aging. 2022 Apr 27;3:861761. doi: 10.3389/fragi.2022.861761 (PMC9261439; doi:10.3389/fragi.2022.861761)

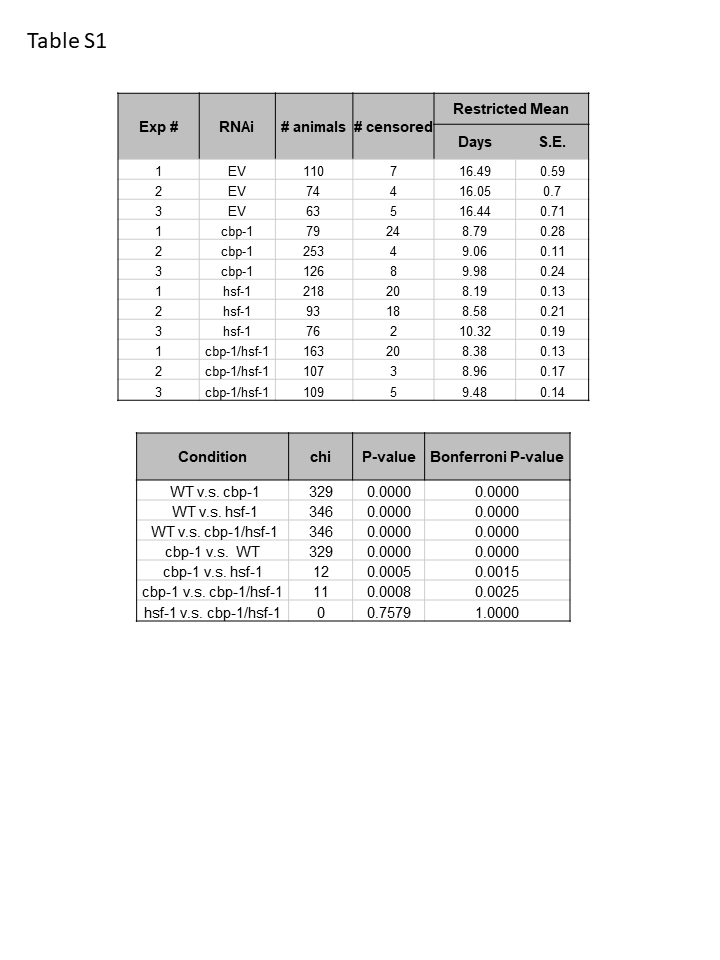

Supplement: Supplementary file 1 [file Image3.TIF]

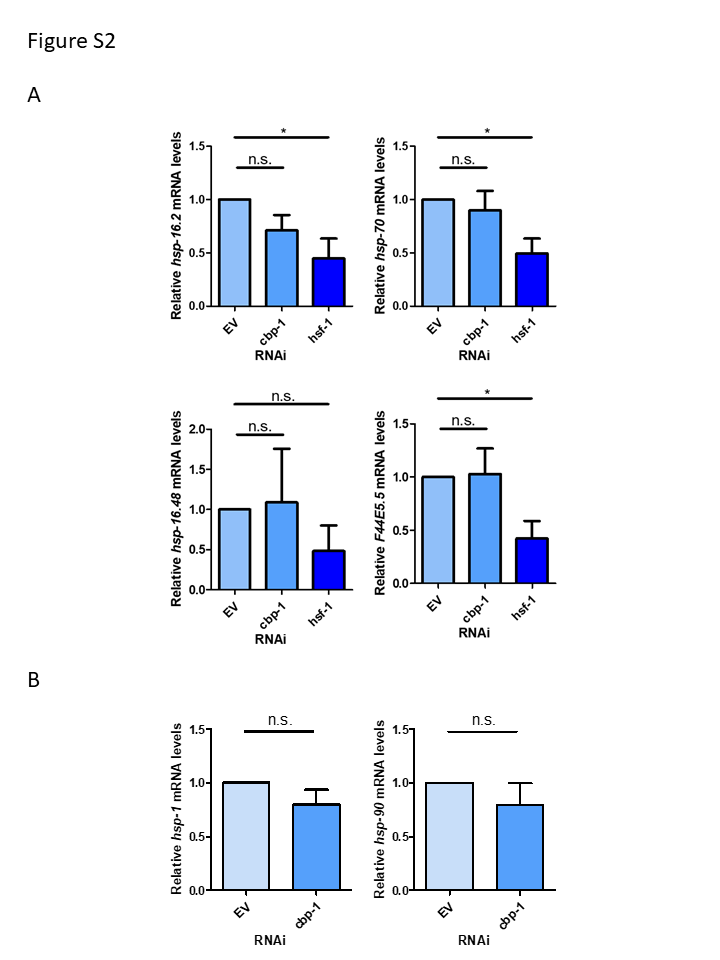

Supplement: Supplementary file 2 [file Image2.TIF]

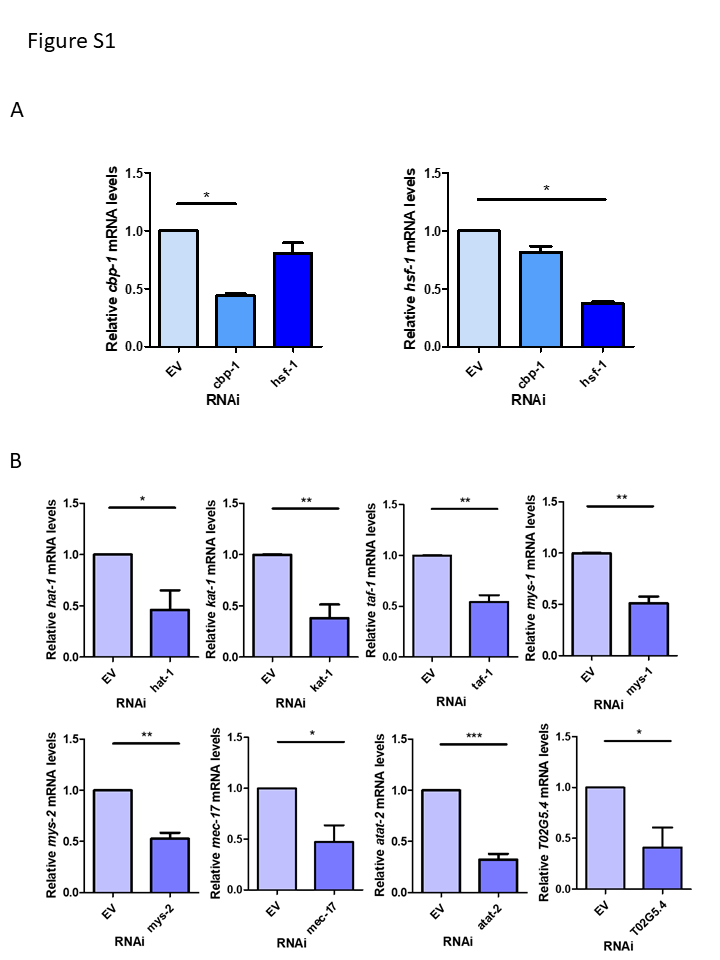

Supplement: Supplementary file 3 [file Image1.TIF]
